# Intestinal Obstruction and Ileocolic Fistula due to Intraluminal Migration of a Gossypiboma

**DOI:** 10.1155/2016/3258782

**Published:** 2016-02-18

**Authors:** Evangelos Margonis, Dionysia Vasdeki, Alexandros Diamantis, Georgios Koukoulis, Grigorios Christodoulidis, Konstantinos Tepetes

**Affiliations:** ^1^Department of Surgery, University Hospital of Larissa, Mezourlo, Larissa, 41110 Thessaly, Greece; ^2^General Surgery, Faculty of Medicine, University of Thessaly, 41500 Larissa, Greece

## Abstract

Gossypiboma refers, as a term, to a retained surgical sponge. It is considered as a rare surgical complication which can occur despite precautions. We report a case of a 36-year-old woman who was admitted to our surgical department with symptoms of abdominal pain associated with episodes of nausea and vomiting that lasted for 2 months. Six months ago she had undergone a cesarean section in a private clinic. Computed tomography revealed a high-density mass occupying a portion of the intestinal lumen, which was reported as a “calcified parasite.” The patient was subjected to laparotomy. The intraoperative findings included signs of obstructive ileus and ileosigmoid fistula and a large sponge was found at the resected portion of the small intestine. Although gossypiboma is a rare entity, it should be included in the differential diagnosis.

## 1. Introduction

Gossypiboma is a medical term that has been used to describe a serious postoperative complication. A retained gauze/sponge in the abdominal cavity after a surgical procedure has been mainly related with this entity. Terms that have also been used to describe this situation are textiloma, cottonoid, cottonballoma, or gauzeoma [1]. The term gossypiboma derives from the Latin word “gossypium” and the Swahili word “boma,” which mean cotton and a “place of concealment,” respectively [2]. The term textiloma originates from the word textile which describes the main material of the innocent foreign bodies (gauzes, sponges, and swabs) that are mainly used intraoperatively. When a gauze is accidentally left in an abdominal cavity, then the patient may experience what has been described as intra-abdominal gossypiboma. Intra-abdominal gossypiboma may present with a variety of gastrointestinal manifestations, depending on the location of the retained gauze in the abdomen, with transmural migration of the gauze being a rare manifestation of it. We present the case of an ileosigmoid fistula and small intestine obstruction secondary to intraluminal migration of a sponge left in the abdominal cavity after a cesarean section.

## 2. Case Report

**Figure 1:**
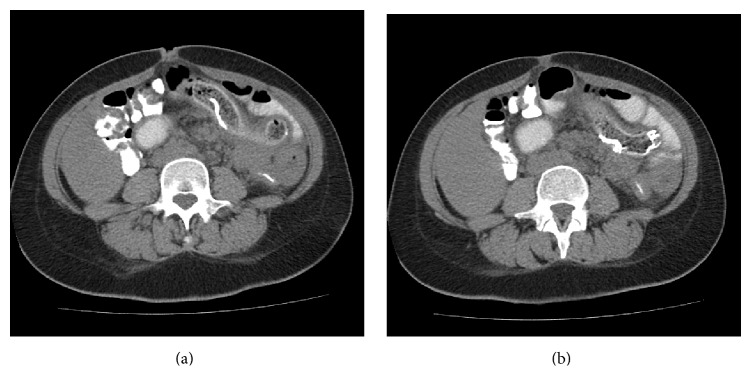
CT images revealing a calcified object inside bowel lumen.

A 36-year-old woman was admitted to our surgical department with abdominal pain associated with episodes of nausea and vomiting over the last couple of months. She denied any history of fever, while she reported a weight loss of around 6 kilos over the same period. She also mentioned that she had undergone a cesarean section in a private clinic 6 months ago. There were no significant findings from the clinical examination of the abdomen. Before having been reviewed by our team, the patient consulted two different general practitioners. The first one set the diagnosis of viral gastroenteritis, while the second advised her to have a computed tomography (CT) scan of her abdomen. The scan was carried out on outpatient basis and revealed a longitudinal, high-density mass inside the intestinal lumen (Figures 1(a) and 1(b)), which was reported as a “calcified parasite.”

**Figure 2:**
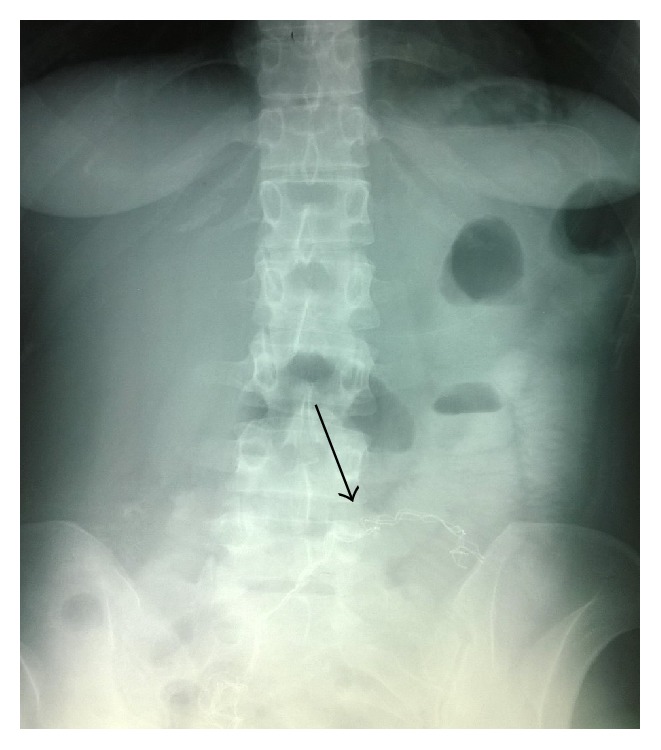
Plain abdominal radiograph revealing a gauze in abdominal cavity.

Subsequently, the patient was transferred to our unit. As an inpatient, she had further imaging studies: an abdominal sonography that was normal and a plain abdominal radiograph which revealed a high-density mass in the left iliac fossa mimicking a retained radiopaque body (Figure 2).

**Figure 3:**
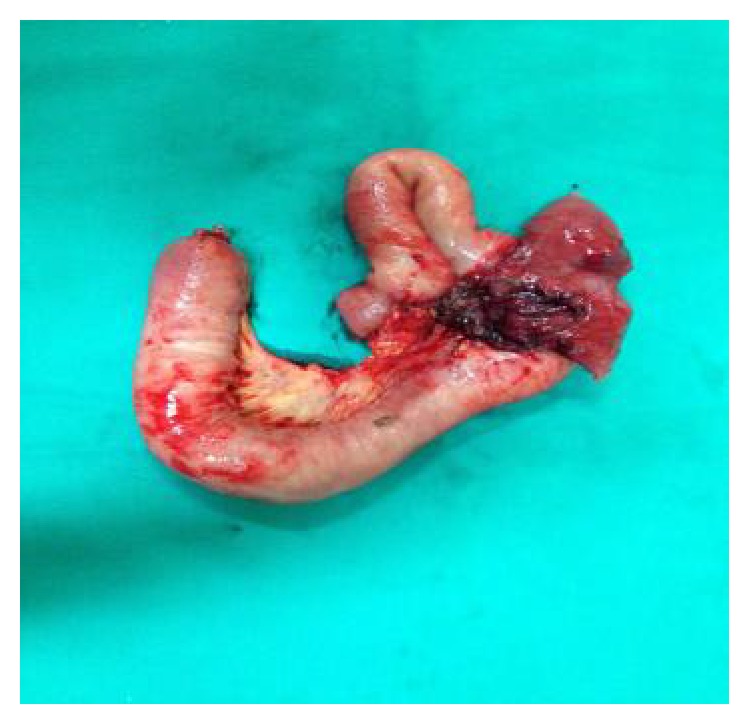
Image of the small bowel resection en bloc with part of affected sigmoid colon.

**Figure 4:**
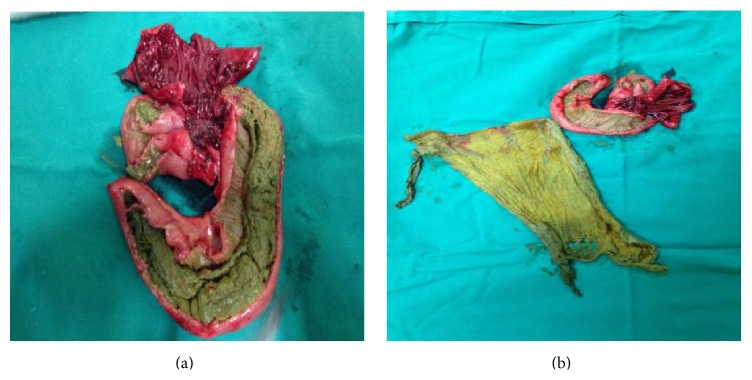
Images of the resected specimen showing the retained foreign body.

Blood tests were within normal values. The case was discussed with the patient and her family and a decision was made for the patient to have an explorative laparotomy. Intraoperative findings included dilatation of the small intestine, secondary to obstruction, material in the lumen of an ileal loop, and an ileosigmoid fistula. A small bowel resection en bloc with the affected part of the sigmoid (Figure 3) and a loop sigmoidostomy were carried out. The small intestine was dissected and a 20 × 25 cm sponge was found in its lumen (Figures 4(a) and 4(b)). The histological findings are consistent with acute and chronic inflammation and fibrosis of pericolic fat. Liponecrosis is also confirmed, with the presence of histiocytes and multinucleated giant cell producing foreign material phagocytosis.

The patient had an uneventful recovery and was subsequently discharged on the eighth postoperative day. Two months later the patient had a successful restoration of the bowel continuity following a minimally invasive closure of the loop sigmoidostomy.

## 3. Discussion

The first case of a retained cotton-matrix item, a gossypiboma, was described by Wilson in 1884 [3]. The abdominal cavity is the most common site where these masses can be found [4]. There have been described several cases of foreign bodies left in the breast, the nervous system, and the thorax [5]. Although a surgical sponge is the most common retained material in the abdomen following a laparotomy, doctor glasses, light bulbs, porcupine quills, and soft drink bottles have also been reported to have been left in the abdominal cavity [6]. The incidence of gossypiboma varies between 1 in 1000 to 1500 of all intra-abdominal operations and 1 in 100 and 1 in 3000 for all surgical procedures [1, 7–9]. Although this frequency is more likely to be underestimated, the fear of adverse publicity and legal implications leads to underreporting of diagnosed gossypibomas [10]. Serious medicolegal problems may arise between the patient and the surgeon, when a diagnosis of gossypiboma has been set. This conflict may have variable consequences for the surgeon, such as wide negative media coverage, humiliation, mental agony, loss of reputation, and litigation [11]. A retained gauze in the abdominal cavity can cause two types of foreign body reaction: either an exudative reaction, causing an abscess formation, or a foreign body granuloma created by an aseptic fibrinous response [12]. Transmural migration of a gauze is rarely reported in the literature and can lead to fistula formation with adherent organs or even intestinal obstruction. The foreign body migration is attributed to the pressure of the necrosed bowel wall, which has been caused by an encapsulated segmental bowel loop. This encapsulation is the result of the inflammatory reaction of the peritoneum due to the presence of foreign material [12, 13]. Small bowel and mainly ileum loops are the most commonly affected parts of the gastrointestinal tract, while there have been reported a few cases of colon and stomach involvement [7]. Cholecystectomy has been most commonly associated with transmural migration cases, followed by cesarean section and hysterectomy [7]. Preventing the retention of a foreign body is much more crucial than curing the condition which is associated with its retention. Sponge counting protocols must be strictly followed in every operating room. In most hospitals one or two members of the operating team, more usually the nurses, routinely perform a proper sponge counting. All materials and equipment should be counted once at the beginning of an operation and twice at the end of it. The surgeon must explore the abdominal cavity or any other operation site and should never close the wound if an incorrect sponge count exists. Although preventing through counting has been suggested to be the “best treatment,” it has been described that most cases occurred although a correct sponge/gauze count was performed. Up to 88% of reported cases with retained material were associated with a correct surgical material counting before wound closure [14]. Furthermore the use of radioopaque sponges has been related with a reduction in the incidence of this entity and their use should be encouraged [15].

## 4. Conclusion

Intra-abdominal gossypiboma is a rare postoperative complication with potentially severe gastrointestinal manifestations and in most cases the diagnosis may be delayed. The intraluminal location of the retained body has been extremely rarely reported, especially as a cause of intestinal obstruction. Prevention measures and precautions should be our primary goal to avoid such an event, which is associated with significant morbidity and medicolegal implications. The possibility of an existing gossypiboma should always be kept in the physician's mind, when a patient presents with abdominal pain following a recent abdominal surgery.
